# Preclinical and Limited Clinical Evidence for Spirulina in Ulcerative Colitis: A Systematic Review and Meta-analysis

**DOI:** 10.34172/apb.025.46031

**Published:** 2025-08-18

**Authors:** Khadije Gorgi, Zahra Ghanbarzadegan, Amir Darkhosh, Sara Shojaei-Zarghani, Seyed Vahid Hosseini

**Affiliations:** ^1^Colorectal Research Center, Shiraz University of Medical Sciences, Shiraz, Iran; ^2^School of Medicine, Shiraz University of Medical Sciences, Shiraz, Iran

**Keywords:** Spirulina, Colitis ulcerative, Systematic reviews as topic, Oxidative stress, Inflammation

## Abstract

**Purpose:**

Ulcerative colitis (UC) is a chronic inflammatory disease of the gastrointestinal tract. This study aimed to systematically review available animal and clinical studies on the effects of spirulina (*Arthrospira platensis*), a natural anti-inflammatory and antioxidant agent, on the condition of UC.

**Methods:**

We conducted a systematic search in the PubMed, Scopus, Web of Science, and Embase databases for studies published from 1980 to April 2024. Experimental studies involving animal (mammalian) models or patients with UC were included. Pooled effect sizes were reported as mean differences (MD) or standardized mean differences (SMD) and 95% confidence intervals (CIs).

**Results:**

A total of 1,321 documents were identified through the systematic search. Following screening, 16 animal studies and 3 randomized controlled trials (RCTs), derived from one trial, were included. The beneficial effects of spirulina on body weight (MD=8.61, 95% CI=2.98 to 14.25, I^2^: 99.78%), clinical features (SMD=-2.39, 95% CI=-2.95 to -1.83, I^2^: 5.89%), colon length (MD=1.25, 95% CI=0.59 to 1.91, I^2^: 95.80%), oxidative stress, inflammatory markers, and gut microbiota in animal models of UC were reported. However, no effect of spirulina on disease activity was reported in the only RCT conducted. Nonetheless, improvements in quality of life, oxidative stress, sleep disturbances, stress scores, and anemia were noted.

**Conclusion:**

Available animal studies suggest beneficial effects of spirulina on UC; however, the limited number of RCTs precludes definitive conclusions.

## Introduction

 Ulcerative colitis (UC) is a prominent subtype of inflammatory bowel disease (IBD) characterized by chronic inflammation of the gastrointestinal tract, particularly affecting the rectum, sigmoid colon, or the entire colon.^1,2^ In 2019, there were approximately 5 million cases of inflammatory bowel diseases (IBD) worldwide, with UC being more prevalent among adults within the spectrum of IBD.^3^ The etiology of UC is multifactorial involving factors such as genetic predisposition, environmental factors, infection, oxidative stress, epithelial barrier defects, dysbiosis, and impaired immune responses.^4^ Major symptoms of UC include bloody diarrhea, often accompanied by mucus, abdominal pain, and weight loss, which typically progress gradually. The management of the disease commonly involves the use of sulfasalazine, 5-aminosalicylates, glucocorticoids, thiopurines, or biologic agents.^5^ Nonetheless, a subset of UC patients turns to complementary and alternative therapies in search of improved symptom control and quality of life.^6^ Consequently, research interest in the effects of herbal remedies on IBD is increasing.

 Spirulina, a safe and easily-digested blue-green alga and filamentous cyanobacterium found in fresh and marine waters, is rich in protein, phenolic acids, γ-linolenic acid, vitamins, and minerals.^7^ It has been reported to possess anti-inflammatory, antioxidant, immunomodulatory, and gut microbiota-promoting properties, suggesting potential effects for UC.^8,9^ However, despite these promising mechanisms, the evidence regarding the impact of spirulina on UC remains conflicting,^9-11^ and no previous systematic review study has been conducted in this regard. Therefore, a systematic review was warranted to comprehensively evaluate the current evidence on the effects of spirulina on UC (animal and human studies), pool data using meta-analysis, and critically appraise the available literature.

## Methods

###  Research strategy

 The present systematic review was registered in the International prospective register of systematic reviews (PROSPERO, ID: CRD42024601732) and conducted according to the PRISMA 2020 guidelines and the Cochrane Collaboration Handbook for Systematic Reviews of Interventions.^12^ A comprehensive systematic search was performed in April 2024 across the PubMed, Scopus, Web of Science, and Embase databases. The search terms were derived from related articles and MeSH terms. The complete search strategy for each database is detailed in Table S1. To ensure that all relevant studies were retrieved, searches in Google Scholar and in the citations and references of all included studies were also conducted. No language restrictions were imposed on the inclusion of studies.

###  Eligibility criteria

 Studies were included if they focused on animal (mammalian) models of UC or on patients suffering from this condition (population). The intervention of interest in the current study was natural whole spirulina (*Arthrospira platensis*, *A. fusiformis*, and *A. maxima*.) or its extracts, administered alone or in combination with standard treatments. Studies that administered spirulina in combination with other agents or its synthetic forms and isolated components *—*rather than the whole alga*—* were excluded to focus on the effects of natural whole spirulina, thereby reflecting its real-world applications in nutritional contexts. Animal studies were included if the comparator was water, a vehicle, saline, no treatment, or standard treatment. Proteins of interest were included in the meta-analysis only if they were reported as absolute concentrations rather than relative expression levels. This approach ensured methodological consistency and enhanced clinical interpretability. Human studies were included only if they had a placebo control group. Disease activity was considered the primary outcome of the present study (Table S2).

###  Study selection and data extraction 

 All retrieved documents were exported to EndNote version 21. After the removal of duplicates, study selection was conducted by two independent reviewers according to predetermined eligibility criteria. Following the exclusion of certain studies based on title and abstract screening, the full texts of the remaining studies were evaluated to assess eligibility. The reviewers compared their selections, and any discrepancies regarding the inclusion of studies were resolved through discussion.

 Two investigators (SSZ and ZG) independently extracted data using study-specific forms for animal and human trials, which were refined following pilot testing on 20% of included studies to ensure consistency. Any discrepancies were resolved through discussion. For animal studies, the following information was extracted: first author, year of publication, country, type of animals, UC model, number of animals per group, dose and duration of treatment, route of administration, type of control, laboratory techniques, and findings. Mean and standard deviation (SD) were extracted for each quantitative variable to be included in the meta-analysis. If multiple doses of interventions were assessed within a single study, we combined the groups. However, if different forms of spirulina were investigated in one study with a common control group, the sample size of the control group was divided to facilitate independent comparisons.^12^ When data were reported as median with minimum and maximum values, or as median and 25th and 75th percentiles, we calculated the mean and SD using the appropriate formulas.^13^ Additionally, the following information was extracted from randomized controlled trials (RCTs): first author, year of publication, country, population characteristics, sample size, gender distribution, mean age, intervention details (type, dose, route of administration, treatment duration), control group details, outcomes assessed, and findings.

###  Risk of bias (ROB) assessment

 The ROB assessment for the included animal and clinical studies was conducted independently by two trained reviewers (SSZ and ZG) using the Office of Health Assessment and Translation (OHAT)^14^ or Cochrane tools,^12^ respectively. Inter-rater reliability was assessed, demonstrating acceptable agreement (Cohen’s κ = 0.67). The OHAT tool considers six types of bias for each individual study: selection bias, performance bias, attrition bias, detection bias, selective reporting bias, and other sources of bias. Each question in the tool was assigned a numerical value: -2 (definitely high ROB), -1 (probably high ROB), + 1 (probably low ROB), and + 2 (definitely low ROB). Subsequently, studies were classified as having low, moderate, or high ROB based on their average scores.^15^ The Cochrane tool also considers similar biases.

###  Statistical analysis

 Statistical analyses were conducted using Stata MP Version 16. Pooled effect sizes were estimated using a random-effects model and expressed as mean differences (MD) or standardized mean differences (SMD), depending on the comparability of outcome scales across studies, with 95% confidence intervals (CI). Between-study heterogeneity was assessed using Cochran’s Q test and I^2^ statistics.^12^ Additionally, funnel plot asymmetry and Egger’s test were applied to assess publication bias when more than 10 studies were included in the analysis. The trim-and-fill analysis was also performed to address potential publication bias. Subgroup analysis was also conducted to determine the sources of heterogeneity.* P* values of less than 0.05 were considered statistically significant.

## Results

###  Study selection

**Figure 1:**
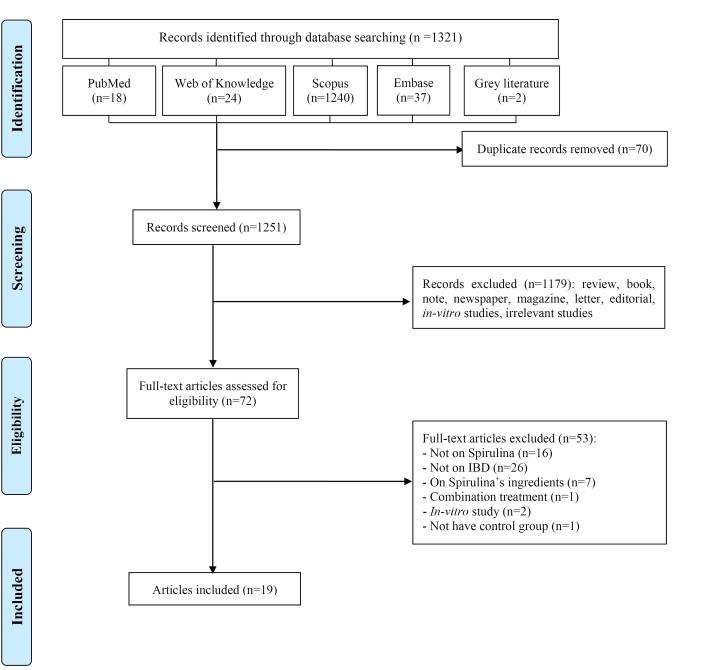
PRISMA flow diagram

 Through a comprehensive search of databases and grey literature, a total of 1,321 documents were retrieved. After the removal of duplicates (n = 70) and the exclusion of certain studies based on title and abstract screening, the full texts of 72 articles were evaluated for eligibility. Ultimately, 16 animal studies and 3 RCTs were deemed eligible and included in the current systematic review. The study selection process is illustrated in Figure 1.

###  Study Characteristics

 Half of the included animal studies focused on rats,^9,10,16-21^ while the other half involved mice.^22-29^ UC was induced in the animals through intrarectal administration of acetic acid (AA)^10,16,18,20,21^ or trinitrobenzene sulfonic acid (TNBS),^19,27^ or via oral administration of dextran sulfate sodium (DSS).^9,22-25,27-29^ One study employed a combination of both AA and DSS for UC induction.^17^ Burkhardt et al employed interleukin-10 knockout (IL-10^-/-^) mice, which serve as a genetically engineered model for UC.^26^ Except for one study that utilized a diet containing 20% spirulina, the remaining studies administered it intragastrically at doses ranging from 50 mg/kg^28^ and 2 g/kg^19^ of body weight; however, the exact dose of spirulina was not reported in four studies.^23-25,27^ Spirulina was administered in various forms: whole, hydroalcoholic extract, aqueous extract, chloroform extract, or fermented form. The duration of spirulina administration varied from three days^27^ to eight weeks.^16^ With the exception of one study that did not employ a placebo control,^17^ the others used water, saline, phosphate-buffered saline, or a vehicle as controls. In addition to placebo controls, four studies included routine treatments for UC as active control groups (Table 1).^9,10,18,20^

**Table 1:** Characteristics of the included animal studies on the effects of spirulina on ulcerative colitis

**First author**	**Year, Country**	**Animals**	**UC model**	**n/group**	**Treatment and dose**	**Study duration**	**Control**	**Effects of spirulina** *** vs*** **. control**
Arrari F^16^	2024, Tunisia	Male Wistar rats	A. obesogenic diet + AA at 59^th^ day, enema; B. standard diet + AA	7	Spirulina, 500 mg/kg, i.g. for 8 weeks	60 days	Distilled water	Reduction of weight gain and adipose tissue; Protection against AA effects on colon weight/length, serum and colonic calcium and free iron, histopathological and morphological features, weight to length ratio, colonic oxidative stress and lipid peroxidation
Zhong D^24^	2024, China	Male C57BL/6J mice	3 cycles of DSS, each consisted of 1 week of drinking DSS followed by two weeks of drinking water	10	Spirulina, 2 mg/mL (0.6 mg/mice), i.g. throughout the 2nd and 3nd cycles of drinking water, every other day, for a total of 14 times	9 weeks	PBS	Reduction of the colonic IL-6 and IL-1β expression; Gut microbiota modulation; Improvement of anxiety; Reduction of plasma S100β and zonulin levels
Wang N^23^	2024, China	Male C57BL/6J mice	DSS in drinking water from 14^th^ to 19^th^ day	8	Non-fermented and fermented spirulina, i.g. from 0 to 19^th^ day	20 days	Water	Protection against DSS effects on weight reduction, shortening colon length, DAI score, and histopathological changes; Modification of gut microbiota, especially by the fermented; Down-regulation of inflammatory cytokines, MyD88, and TLR4 and improvement of integrity of the mucosal barrier, especially by the fermented
Chen QW^27^	2023, China	Female C57BL/6J mice	A. DSS in drinking water from first to 6^th^ day B. TNBS presensitization in the first day and at 7^th^ day TNBS, enema	5	A. Spirulina, 5 mg/mL (1 mg/mice), i.g., at 4^th^, 6^th^, 8^th^, 10^th^, and 12^th^ days B. Spirulina, 5 mg/mL (1 mg/mice), i.g., at 8^th^, 9^th^, and 10^th^ days	A. 14 days B. 11 days	PBS	Prevention of colonic shortening and weight loss; Reduction of IL-6 and TNF-α and elevation of IL-10; Increasing the expression of ZO-1 and claudin-1; Improvement of histopathological features
Wang J^22^	2022, China	C57BL/6 mice	DSS in drinking water for 8 days (from 1st to the 8^th^ day)	10	Spirulina aqueous extract, 300 mg/kg, i.g. (from 0 to 11^th^ day)	11 days	1. vehicle; 2. sulfasalazine, 300 mg/kg, i.g.	Prevention of colonic shortening, weight loss, the increase in DAI score, histopathological injuries, MPO level, changes of inflammatory and anti-inflammatory cytokines in the serum and colon, and oxidative stress; Improvement of tight junction and inflammatory proteins and gut microbiota; The effects were comparable with sulfasalazine, or even better.
Yacoub MB^17^	2022, Egypt	Male albino rats	Single dose of AA via enema + DSS in drinking water for 7 days	8	Spirulina, 500 mg /kg, i.g., for 7 days	7 days	No treatment	Prevention of weight loss; Improvement of clinical and histopathological features; Reduction of MDA, TNF-α, and IL-1β; Increase in CAT and SOD
Burkhardt W^26^	2021, Germany	Male C57BL/6.129P2-Il10^tm1Cgn^ mice	IL10-deficient (IL-10^-/-^) mice model	12	Diet containing 20% spirulina powder	3 weeks	Isocaloric control diet	Elevation of histopathological score and cecal TNF-α expression; Improvement of gut microbiota
Zhong D^25^	2021, China	Female Balb/c mice	DSS for 10 days (from 0 to 10^th^ day)	5	Spirulina, 1.7 mg/mL (0.43 mg/mice), i.g. from 4^th^ to 16^th^ day	16 days	PBS	Reduction of rectal bleeding and weight loss; Improvement of colon length, oxidative stress, and histopathological score; Reduction of the TNF-α and IL-6 expression
de Oliveira Garcia FAO^28^	2020, Brazil	Male C57BL/6 mice	DSS for 6 days	6	Spirulina extract, 50, 100, 250 mg/kg, i.g. for 5 days	6 days	Saline	Dose-dependent improvements in the clinical signs and weight loss; Protection against DSS-induced histopathological changes (at all doses) and inflammation (especially at doses of 50 and 250)
Guo W^29^	2019, China	Male C57BL/6 mice	DSS for 4 days (from 0 to 3^th^ day)	8	Aqueous extracts of spirulina, 200mg/kg, for 8 days (from 0 to 7^th^ day), i.g.	8 days	PBS	Prevention of body weight and colon length reduction; Improvement of DAI and histological lesions; Increment of HSP-25, claudin-4 and occluding and reduction of COX-2, iNOS, MPO, TNF-α, IL-1β, and IL-6
Morsy MA^9^	2019, India	Male Wistar rats	DSS in drinking water	6	Chloroform or hydroalcoholic extracts of spirulina, 100 and 200 mg/kg/day simultaneously with DSS, i.g.	15 days	1. vehicle; 2. sulfasalazine, 50 mg/kg, i.g.	Dose-dependent protection against DSS-induced loss of body weight; Dose-dependent improvement of DAI score by hydroalcoholic extracts; Dose-dependent modulation of DSS-induced inflammatory changes especially by hydroalcoholic extracts; Dose-dependent reduction of MPO activity by hydroalcoholic extracts; Improvement of histopathological features by high dose of hydroalcoholic extracts
Ghazy EW^20^	2019, Egypt	Male albino rats	AA, enema, at 10^th^ day	8-9	Spirulina, 500 mg/kg, i.g. from the 1st to the 10^th^ day	15 days	1. Saline, 2. Mesalazine, 20 mg/kg, i.g., from 10^th^ to 15^th^ day	Prevention of colonic shortening and weight loss, DAI, macroscopic damages, and histopathological features; Reduction of MDA, NO, iNOS, COX-2 The effects were almost comparable with mesalazine, or even better
Rezaei N^10^	2019, Iran	Male SD rats	AA, enema, at the first day	8	Spirulina, 1 g/kg, i.g. from first to 7^th^ day	7 days	1. Normal saline 2. Mesalazine (100 mg/kg) 3. Sulfasalazine (360 mg/kg)	Protection against AA-induced colonic shortening and elevation of inflammatory cytokines, MPO levels, colonic MDA and PGE2 levels; Elevation of antioxidant agents; Improvement of colonic mucosa changes The effects were almost comparable with sulfasalazine and mesalazine
Rezaei N^21^	2018, Iran	Male SD rats	AA, enema, at 30^th^ day	8	Spirulina, 1 g/kg, i.g. from the 1st to the 32nd day	32 days	Normal saline	Protection against AA-induced colonic shortening and elevation of inflammatory cytokines, MPO levels, colonic MDA and PGE2 levels; Elevation of antioxidant agents; Improvement of colonic mucosa changes
Abdel-Daim MM^18^	2015, Egypt	Male Wistar albino rats	AA, enema, at 16^th^ day	8	Spirulina alcoholic extract, 500 mg/kg, i.g., for 15 days	17 days	1. Normal saline; 2. sulfasalazine, 500 mg/kg, i.g., in 13^th^, 14^th^ and 15^th^ days	Prevention of colonic shortening and weight loss; Improvement of bloody diarrhea, oxidative stress, lipid peroxidation, MPO activity, inflammatory and immunomodulatory markers, and histopathological features. The effects were almost comparable with sulfasalazine, or even better.
Coskun ZK^19^	2011, Turkey	Wistar rats	TNBS, enema	8	Spirulina, 2 g/kg, i.g. for 7 days after TNBS administration	8 days	Normal saline	Prevention of weight loss; Reduction of tissue MDA; Improvement of histopathological features; Reduction of apoptosis

AA: acetic acid; CAT: catalase; COX: cyclooxygenase; DAI: disease activity index; DSS: dextran sulfate sodium; HSP: heat shock proteins; i.g.: intragastric; IL: interleukin; iNOS: inducible nitric oxide synthase; MDA: malondialdehyde; MPO: myeloperoxidase; MYD: Myeloid differentiation primary response; NO: nitric oxide; PBS: phosphate-buffered saline; PGE2: prostaglandin E2; S100β: S100 calcium-binding protein beta subunit; SD: Sprague Dawley; SOD: superoxide dismutase; TLR: toll-like receptor; TNBS: trinitrobenzene sulfonic acid; TNF-α: tumor necrosis factor-α; UC: ulcerative colitis; ZO: zonula occludens.

 Only one RCT investigated the effects of spirulina on patients with UC, from which three studies were derived, each reporting non-overlapping outcomes. This trial was conducted in Iran and involved 73 patients suffering from active mild to moderate UC (48% male), with a mean age of 38.64 years (Table 2).^11,30,31^

**Table 2:** Characteristics of the included clinical trials on the effects of spirulina supplementation on ulcerative colitis

**First author**	**Year**	**Country**	**Sample size (spirulina group)**	**Men (%)**	**Age (year),** **Mean±SD**	**BMI (kg/m**^2^**),** **Mean±SD**	**Dose**	**Treatment duration**	**Control**	**Outcomes**	**Effects of spirulina ** ***vs*** **. control**
Moradi S^11^	2024	Iran	73 (36)	48	38.64 ± 11.30	25.81 ± 4.96	1 g/day (bid)	8 weeks	Corn starch without chlorophyll	Anthropometry, disease activity, health-related quality of life, serum MDA, TAC, SOD, ESR, pentraxin-3	↑ TAC ↑ Quality of life
Moradi S^30^	2023	Iran	73 (36)	48	38.64 ± 11.30	25.81 ± 4.96	1 g/day (bid)	8 weeks	Corn starch without chlorophyll	CBC, serum iron, ferritin, fecal occult blood test	↑ Serum iron ↑ RBC ↑ HCT ↓ MCV
Moradi S^31^	2021	Iran	73 (36)	48	38.64 ± 11.30	25.81 ± 4.96	1 g/day (bid)	8 weeks	Corn starch without chlorophyll	Blood pressure, sleep quality, mental health, fatigue status	↓ Sleep disturbances and stress score

bid: twice a day; CBC: complete blood count; ESR: erythrocyte sedimentation rate; HCT: hematocrit; MCV: mean corpuscular volume; MDA: malondialdehyde; RBC: red blood cell; SD: standard deviation; SOD: superoxide dismutase; TAC: total antioxidant capacity

###  ROB assessment

 The results of the ROB assessment for the included animal studies are presented in Table S3. In four of the included studies, no data about randomization were reported,^16,18,21,26^ and one study employed a non-treated control group.^17^ The sex of the animals was not specified in two of the included studies.^19,22^ Blinding is a critical factor in both the administration of interventions and histopathological assessments, particularly in studies involving components other than natural spirulina as the primary focus or those examining multiple doses; however, this aspect was not addressed in several of the included studies. Finally, with the exception of four studies,^16,17,19,21^ the remaining studies were classified in the first tier (low ROB). The results of the ROB assessment for the included RCTs are also reported in Table S4.

###  Outcomes

####  Animal studies

#####  a. Clinical features 

**Figure 2:**
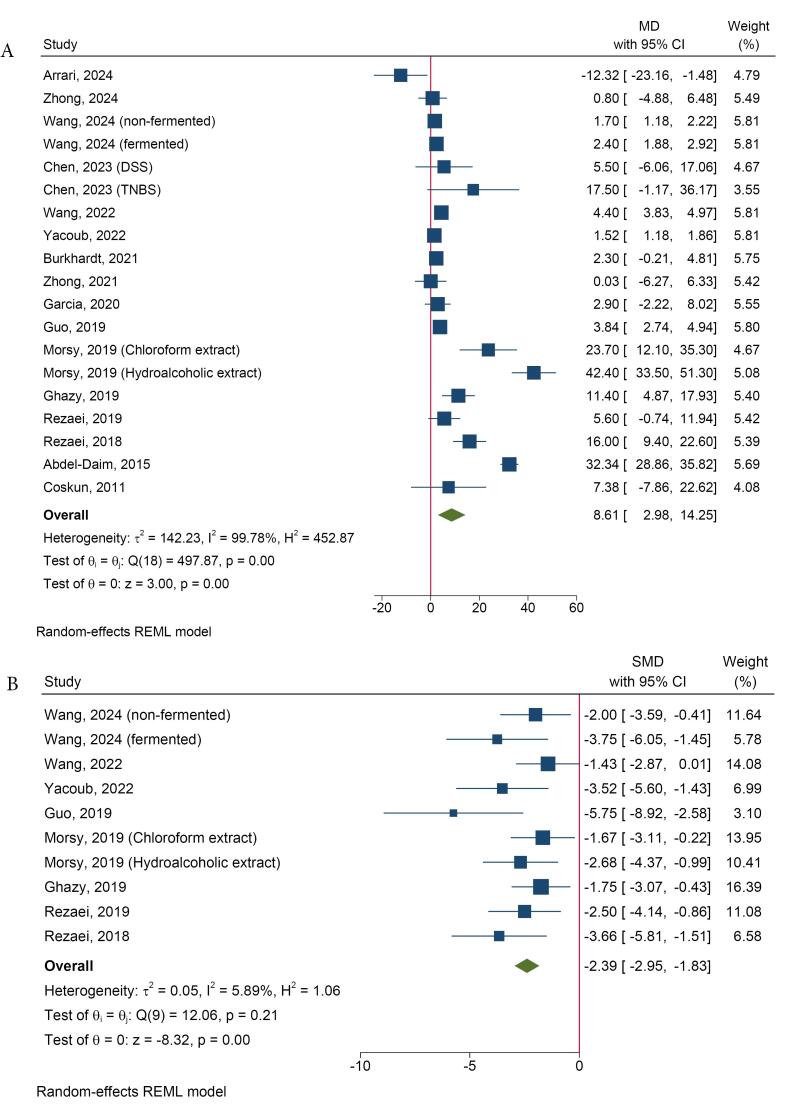
Forest plots depicting the effects of spirulina on body weight (A) and disease activity index (B) in ulcerative colitis

 All included studies assessed the effects of spirulina on body weight in various animal models of UC. Spirulina significantly attenuated UC-induced weight loss (MD = 8.61, 95% CI = 2.98 to 14.25, I^2^: 99.78%) (Figure 2A). The method of UC induction was identified as a source of heterogeneity in this analysis (Table S5).

 Several of the included studies evaluated the clinical features of the animals using the Disease Activity Index (DAI), which was calculated as the sum of scores for body weight loss, stool consistency, and fecal bleeding.^9,10,17,20-23,29^ Meta-analysis showed that spirulina reduced DAI scores relative to controls (SMD = -2.39, 95% CI = -2.95 to -1.83, *I*^2^: 5.89%) (Figure 2B). Furthermore, de Oliveira Garcia et al included parameters such as wet anus, bleeding stools, stool consistency, piloerection, and hypoactivity as clinical indices and reported beneficial effects of spirulina on the overall clinical score.^28^ Three other studies also assessed the effects of spirulina on alleviating bleeding associated with UC in animals,^18,24,25^ with two studies reporting a beneficial effect.^18,25^

#####  b. Colonic morphology

**Figure 3:**
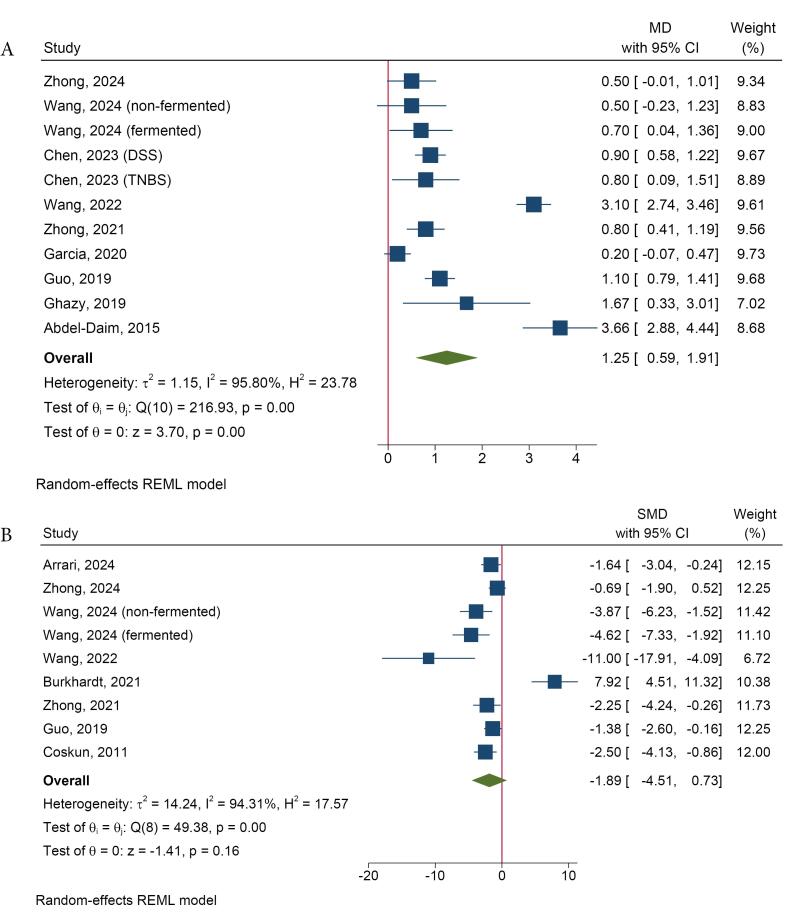
Forest plots depicting the effects of spirulina on colon length (A), and histopathological score (B) in ulcerative colitis

 The pooled effect analysis indicated that spirulina significantly prevents the shortening of colon length in animal models of UC (MD = 1.25, 95% CI = 0.59 to 1.91, *I*^2^: 95.80%) (Figure 3A). The form of administered spirulina (whole or extract) was identified as a source of heterogeneity in this analysis (Table S5). Most studies reported that spirulina reduced colon weight and the weight-to-length ratio, counteracting UC-induced changes.

 Some studies have also examined additional morphological characteristics of colonic tissue in the UC and the impact of spirulina on these features. Arrari et al conducted a macroscopic evaluation of colonic tissues in animals, focusing on mucosal erythema, mucosal edema, bleeding ulcers, and tissue necrosis. They reported a beneficial effect of spirulina in inhibiting AA-induced injuries.^16^ In two other studies by Ghazy et al^20^ and Abdel-Daim el al,^18^ spirulina was found to prevent AA-induced colonic edematous inflammation, adhesion, and other macroscopic damages.

#####  c. Histopathological features

 UC was characterized by significant histopathological alterations, including extensive inflammatory cell infiltration and congestion of epithelial cells within colonic fragments. The disease resulted in severe damage to the intestinal epithelium, leading to disrupted crypt architecture, loss of goblet cells, mucosal and submucosal ulceration, necrosis, hemorrhage, and edema. All studies investigated the effects of spirulina on UC-induced histopathological changes, and except two studies, the remaining studies reported beneficial effects. In 2024, Zhong et al demonstrated no beneficial effects of spirulina against histopathological alterations induced by DSS.^24^ Additionally, the study by Burkhardt et al indicated increased immune cell infiltration into the lamina propria of the cecum in animals treated with spirulina compared to control IL-10 knockout mice.^26^

 Some studies reported levels of histopathological changes, including crypt damage, necrosis, and inflammation, using a quantitative variable referred to as the histopathological score, which employed different scoring systems across the studies. The results of the meta-analysis indicated no significant reduction in the histopathological score with spirulina treatment compared to the positive control group (SMD = -1.89, 95% CI = -4.51 to 0.73, *I*^2^: 94.31%) (Figure 3B). Additionally, there were no significant differences among the various analyzed subgroups. The type of animal and ROB were identified as sources of heterogeneity in this analysis.

#####  d. Oxidative stress

 Except for seven studies,^9,23,24,26-29^ the remaining assessed the effects of spirulina on serum or colonic levels of antioxidants (catalase [CAT],^16-20,22^ superoxide dismutase [SOD],^10,16-18,21,22^ glutathione peroxidase [GPx],^10,16,21,22^ reduced glutathione [GSH],^10,16,18-21^ thiol group,^16^ total antioxidant capacity [TAC],^10,21^ total antioxidant status [TAS]),^19^ scavenging activity,^16^ or oxidative biomarkers (advanced oxidation protein products,^19^ protein carbonyl,^18^ malondialdehyde (MDA),^10,16-22^ free iron,^16^ total reactive oxygen species (ROS), superoxide anion [O_2_^•^-], hydroxyl radical [^•^HO], hydrogen peroxide [H_2_O_2_])^16,25^ in animals with UC. Apart from two studies,^19,20^ others reported beneficial effects of spirulina on all assessed oxidative stress-related biomarkers.

**Figure 4:**
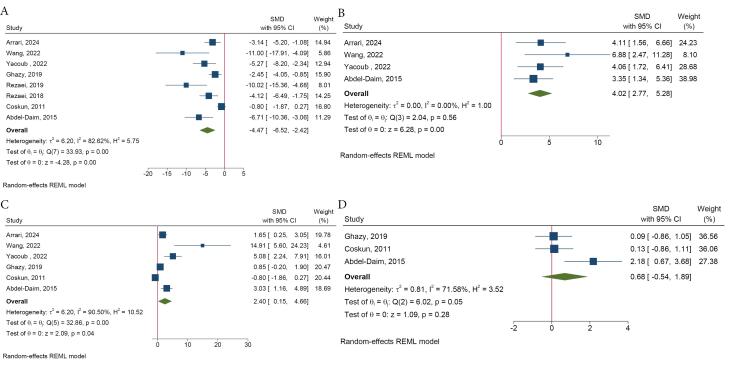
Forest plots depicting the effects of spirulina on colonic oxidative stress, including malondialdehyde (A), superoxide dismutase (B), catalase (C), and reduced glutathione (D) in ulcerative colitis

 According to our meta-analysis, spirulina lowered colonic MDA (SMD = -4.47, 95% CI = -6.52 to -2.42, *I*^2^: 82.62%) and increased colonic SOD (SMD = 4.02, 95% CI = 2.77 to 5.28, *I*^2^: 0.00) and catalase levels (SMD = 2.40, 95% CI = 0.15 to 4.66, *I*^2^: 90.50%) (Figure 4, A-C). The reduction of colonic MDA was significantly greater with spirulina extract compared to its whole form (SMD = -7.81, 95% CI = -11.48 to -4.14 *vs*. SMD = -3.54, 95% CI = -5.42 to -1.65, *P*-value for difference = 0.04). The form of spirulina was also identified as a source of heterogeneity in the MDA analysis (Table S6). Spirulina had no significant effects on colonic GSH (SMD = 0.68, 95% CI = -0.54 to 1.89, *I*^2^: 71.58%) (Figure 4D).

#####  e. Inflammatory markers

**Figure 5:**
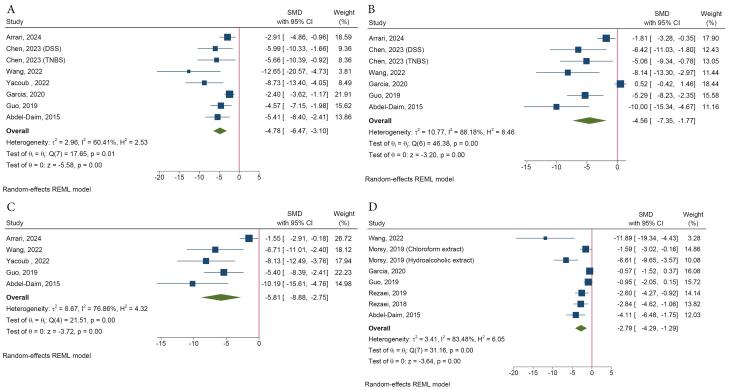
Forest plots depicting the effects of spirulina on colonic inflammatory markers, including tumor necrosis factor-α (A), interleukin-6 (B), interleukin-1β (C), and myeloperoxidase (D) in ulcerative colitis

 Meta-analysis indicated that spirulina reduced colonic tumor necrosis factor-alpha (TNF-α) (SMD = -4.78, 95% CI = -6.47 to -3.10, *I*^2^: 60.41%), interleukin (IL)-6 (SMD = -4.56, 95% CI = -7.35 to -1.77, *I*^2^: 88.18%), IL-1β (SMD = -5.81, 95% CI = -8.88 to -2.75, *I*^2^: 76.86%), and myeloperoxidase (MPO) (SMD = -2.79, 95% CI = -4.29 to -1.29, *I*^2^: 83.48%) compared to the UC group (Figure 5). However, the effects of spirulina on the colonic IL-10 (SMD = 1.92, 95% CI = -1.29 to 5.13, *I*^2^: 89.39%) and nitric oxide (SMD = -3.41, 95% CI = -7.94 to 1.11, *I*^2^: 95.13%) were not statistically significant (Figure S1).

 Several studies have also investigated the effects of spirulina on serum levels of cytokines, and reported the beneficial effects on serum TNF-α (SMD = -2.85, 95% CI = -3.93 to -1.78), IL-6 (SMD = -8.66, 95% CI = -16.03 to -1.29, *I*^2^: 97.65%) and IL-1β (SMD = -5.01, 95% CI = -8.78 to -1.23, *I*^2^: 85.39%) (Figure S2). The effect of spirulina on serum TNF-α was significantly greater in the DSS-induced model compared to the AA-induced model (SMD = -3.86, 95% CI = -5.46 to -2.26 *vs*. SMD = -1.86, 95% CI = -2.84 to -0.88, *P*-value for difference = 0.04). The type of animals, form of spirulina, and UC model were identified as sources of heterogeneity (Table S7).

#####  f. Gut microbiota

 Four of the studies included in this review evaluated the effects of spirulina on gut microbiota in the context of UC. All of these studies reported beneficial effects on the improvement of microbial diversity or composition. Wang et al demonstrated that spirulina, particularly in its fermented form, increased α-diversity and the relative abundance of several taxa, including the phylum Proteobacteria, class Alphaproteobacteria, order Acetobacterales, family*Acetobacteraceae, *and the *Lachnospiraceae NK4A136 group.*^23^ In another study by Wang et al, an increase in α-diversity was observed alongside elevated relative proportions of the phylum Firmicutes, as well as the families*Lactobacillaceae* and *Peptostreptococcaceae*. Additionally, the genera *Lactobacillus*, *Romboutsia*, and *Turicibacter* exhibited increased relative abundances, while a decrease in the relative abundance of unidentified *Enterobacteriaceae* was noted. The community structure in this study was found to be similar to that of the normal group.^22^ Burkhardt et al reported an increased relative abundance of the *Porphyromonadaceae*family along with a decrease in the *Desulfovibrionaceae* family, including its associated species *Bilophila wadsworthia,* in the cecal contents of mice treated with spirulina compared to the control group. However, no significant changes in α-diversity were observed in this study.^26^ In another investigation, although species richness (α-diversity) remained unaffected, spirulina attenuated DSS-induced elevations in the relative abundance of *Candidatus Stoquefichus* and *Monoglobus.*^24^

####  Human studies

 In the only RCT, twice-daily 500 mg spirulina capsules improved quality of life, sleep disturbances, stress levels, serum TAC level, and anemia. Conversely, there were no significant effects observed on UC disease activity, anthropometric variables, serum MDA, SOD, erythrocyte sedimentation rate (ESR), pentraxin-3, or blood pressure.^11,30,31^

###  Publication bias

 Publication bias was assessed for three outcomes of interest with > 10 included studies; results are presented in Figure S3. Based on visual inspection, Egger’s test, and Begg’s test, significant publication bias was observed only for DAI (*P* values < 0.001). Trim-and-fill analysis imputed 3 additional studies, changing the pooled SMD to -2.10 (95% CI: -2.73 to -1.47) without significant alteration (Figure S4).

## Discussion

###  Summary of evidence

 The included animal studies suggest that spirulina may exert beneficial effects on UC through positive modulation of body weight, clinical symptoms, inflammatory markers, oxidative stress, and gut microbiota composition. Notably, spirulina in extract form demonstrated significantly greater antioxidant effects than the whole form. Our meta-analyses identified animal type, spirulina form, ROB, and UC model as key sources of between-study heterogeneity. The sole RCT available in this area reported improvements in quality of life, sleep disturbances, stress, oxidative stress, and anemia, but not in UC disease activity. As most findings in this review are derived from animal research, well-designed RCTs are needed to determine whether these results are translatable to humans.

###  Mechanisms 

 Body weight gain is often observed following treatment for UC, which may be partially attributed to improvements in intestinal inflammation, nutritional status, and the catabolic state of the patient.^32^ Animal studies reported beneficial effects of spirulina on body weight gain and clinical features. However, the sole RCT that investigated this did not yield similar findings. Further RCTs should be conducted to draw more definitive conclusions in this regard.

 The anti-inflammatory effects of spirulina in UC may be partly mediated by down-regulation of the toll-like receptor-4 (TLR-4)/myeloid differentiation primary response 88 (MyD88)/nuclear factor-κB (NF-κB) inflammatory signaling pathway.^22,23^ Independently of TLR-4/NF-κB suppression, spirulina may also reduce the expression of cyclooxygenase-2 (COX-2) and inducible nitric oxide synthase (iNOS), thereby modulating downstream cytokine signaling implicated in colonic inflammation in UC.^20,22^ Chronic intestinal inflammation is known to increase oxidative stress, which leads to dysfunction of the antioxidant defense system, damage to cellular biomolecules, mitochondrial dysfunction, impairment of the intestinal epithelial barrier, recruitment of immune cells, and activation of inflammatory pathways. Consequently, these processes can trigger the induction and progression of IBD.^33,34^ Thus, the literature suggests a role for antioxidants in the prophylaxis, management, and treatment of IBD.^35^ While some studies have reported the anti-oxidative effects of spirulina,^36^ not all previous research has reached the same conclusion.^37^ According to the available literature on animal models of UC and the only RCT conducted to date, spirulina may exert beneficial effects on UC by suppressing oxidative stress. However, further clinical studies are warranted in this area.

 The reduction of microbial diversity and gut microbiota dysbiosis have been associated with the pathogenesis of IBD.^38^ The beneficial effects of spirulina on gut microbiota may partly explain its positive impact on UC. Notably, the Firmicutes phylum and the*Lactobacillaceae* and *Lachnospiraceae*families produce butyrate, mitigate inflammation, regulate immune function, and protect epithelial barrier integrity. These groups are depleted in inflamed mucosa,^39-41^ but their abundance increases after spirulina administration in animal models. Further clinical studies are needed to assess the effects of UC and spirulina on human gut microbiota.

 Tight junctions in epithelial cells play a critical role in maintaining cell polarity and the integrity of the intestinal and colonic mucosa. They prevent the diffusion of integral membrane proteins between the apical and basolateral surfaces while regulating paracellular permeability. Structurally, tight junctions consist of transmembrane proteins, such as occludin and claudins, and peripheral membrane proteins like zona occludens. Dysfunction in these junctions has been associated with IBD.^42,43^ Emerging evidence suggests that spirulina may exert beneficial effects on UC by preserving the function of key tight junction proteins, including occludin, claudins, and zona occludens.^22,23^ Additionally, spirulina has been shown to reduce plasma zonulin—a modulator of tight junction permeability—as well as lipopolysaccharide (LPS), a marker of intestinal barrier disruption.^24^ Guo et al also demonstrated that spirulina upregulates cytoprotective heat shock protein 25 (HSP-25; the murine homolog of human HSP-27).^29^ HSPs are known to safeguard the gut epithelium against apoptosis, infection, heat stress, oxidative stress, and inflammation, while also stabilizing the localization of tight junction proteins.^44^

 There are several *in vitro* studies in the literature suggesting the potential effects of spirulina on UC.^22,27,29^ Spirulina has been reported to enhance intestinal barrier function in Caco-2 cells and to exhibit protective effects against hydrogen peroxide (H_2_O_2_)-induced epithelial apoptosis, oxidative stress, and cell membrane damage. Furthermore, it reduced inflammation induced by TNF-α, IL-1β, and H_2_O_2_ in these cells by suppressing the expression of IL-8, COX-2, and iNOS.^29^ In another study, the NCM460 cell line, which consists of normal human colon mucosal epithelial cells, was utilized, and injury was induced using DSS. In this investigation, pretreatment with spirulina improved cell viability, decreased apoptosis and cellular injuries, and lowered intracellular ROS levels.^22^

 Several studies have evaluated the effects of spirulina components on UC. Phycocyanin, a water-soluble pigment-protein complex derived from cyanobacteria, rhodophytes, and cryptophytes,^45^ has been shown to confer protective effects against UC through its antioxidant and anti-inflammatory properties, as well as by enhancing the integrity of the intestinal epithelial barrier.^46^ Additionally, phycocyanin may exert therapeutic effects on UC by improving gut microbiota and downregulating the LPS/TLR4/NF-κB and p38 mitogen-activated protein kinase (MAPK)/MK2 signaling pathways.^47^ Furthermore, microalgal polysaccharides have also been reported to provide protection against DSS-induced colitis.^48^ Future studies should be conducted to more precisely identify the active components of spirulina.

## Limitations

 Our study has several limitations. The meta-analysis was conducted on animal studies, which need to be evaluated in clinical investigations to determine whether these effects are translatable to humans. Additionally, the high level of heterogeneity, the limited number of studies for certain outcomes, and the presence of publication bias represent further limitations of the current study.

###  Future research directions

 Future research should prioritize large, long-term RCTs to evaluate spirulina’s potential in UC management, with particular focus on dose-response relationships and mechanistic interactions with conventional therapies. Additionally, exploratory studies investigating gut microbiota modulation and targeted drug delivery systems could reveal novel applications for UC treatment and prevention.

## Conclusion

 In conclusion, existing animal studies indicate that spirulina may have beneficial effects on clinical features, inflammation, oxidative stress, and gut microbiota dysbiosis. However, the limited clinical evidence precludes definitive conclusions.

## Competing Interests

 None.

## Data Availability Statement

 Data are available from the corresponding author with reasonable request.

## Ethical Approval

 The present study was approved by the Vice-Chancellor for Research and Technology of Shiraz University of Medical Sciences (Ethical ID: IR.SUMS.MED.REC.1403.683).

## Endnotes

 The present study is extracted from the thesis of Amir Darkhosh (Code: 31663).

## Supplementary Files


Supplementary file 1 contains Tables S1-S7 and Figures S1-S4.
