# Supplementary material for: Preclinical and Limited Clinical Evidence for Spirulina in Ulcerative Colitis: A Systematic Review and Meta-analysis
Source: Adv Pharm Bull. 2025 Aug 18;15(4):793–805. doi: 10.34172/apb.025.46031 (PMC12980208; doi:10.34172/apb.025.46031)
Supplement: Supplementary file 1 — contains Tables S1-S7 and Figures S1-S4. [file apb-15-793-s001.pdf]

Adv Pharm Bull. 2025;15(4):793-S1
doi: 10.34172/apb.025.46031
https://apb.tbzmed.ac.ir

Supplementary file 1

Table S1. Search strategy for databases

Database	Search Strategy
**PubMed**	((((idiopathic proctocolitis) OR (colitis gravis)) OR (inflammatory bowel disease*)) OR ((colitis) AND (ulcerative))) AND ((spirulina) OR (arthrospira))
**Scopus**	(((ALL (idiopathic AND proctocolitis) OR ALL (colitis AND gravis) OR ALL (inflammatory AND bowel AND disease*))) OR ((ALL (colitis) AND ALL (ulcerative)))) AND ((ALL (spirulina) OR ALL (arthrospira)))
**Web of Science**	((ALL =(idiopathic proctocolitis) OR ALL =(colitis gravis) OR ALL =(inflammatory bowel disease*) OR ((ALL =(colitis) AND ALL= (ulcerative)))) AND (ALL= (spirulina) OR ALL= (arthrospira)))
**Embase**	((idiopathic AND proctocolitis) OR (colitis AND gravis) OR (inflammatory AND bowel AND disease*) OR (ulcerative AND colitis)) AND (spirulina OR arthrospira)

Table S2. Inclusion and exclusion criteria

PICOS	Inclusion Criteria	Exclusion Criteria
**Population**	Animal models (mammalian) of ulcerative colitis or patients with this disease	Other types of inflammatory bowel diseases or other animals
**Intervention**	Spirulina (*Arthrospira platensis, A. fusiformis, and A. maxima.*) or its extracts, alone or in combination of routine treatment	Combination of spirulina with other agents or synthetic forms; Components of spirulina instead of whole algae
**Comparator**	Placebo, routine treatment	No control group
**Outcome**	The primary outcome was disease activity, and the secondary outcomes were histopathological changes, inflammatory or oxidative markers, microbiota composition, and anthropometric and psychological features	Irrelevant outcomes or those that were not reported as absolute values
**Study design**	Experimental animal or human studies	Review, Note, Editorial, letter, Observational studies, Quasi-experimental studies, in vitro studies

Table S3. The risk of bias assessment and tier classifications of the included animal studies on the effects of spirulina supplementation on ulcerative colitis

CATEGORY	QUESTIONS	Arrari F, 2024[16]	Zhong D, 2024[24]	Wang N, 2024[23]	Chen QW[27]	Wang J, 2022[22]	Yacoub MB, 2022[17]	Burkhardt W, 2021[26]	Zhong D, 2021[25]	Garcia FAO, 2020[28]	Guo W, 2019[29]	Morsy MA, 2019[9]	Ghazy EW, 2019[20]	Rezaei N, 2019[10]	Rezaei N, 2018[21]	Abdel-Daim MM, 2015[18]	Coskun ZK, 2011[19]
Selection Bias	1. Was administered dose or exposure level adequately randomized?	-	+	+	+	+	+	-	+	+	+	+	+	+	-	-	+
	2. Was allocation to study groups adequately concealed?	-	+	+	+	+	+	-	+	+	+	+	+	+	-	-	+
Performance Bias	3. Were experimental conditions identical across study groups?	++	++	++	++	-	- -	++	++	++	++	++	++	++	++	++	-
	4. Were the research personnel and human subjects blinded to the study group during the study?	-	-	-	-	-	-	+	-	-	-	-	-	-	-	-	-
Attrition/ Exclusion Bias	5. Were outcome data complete without attrition or exclusion from analysis?	++	+	++	+	+	++	+	++	+	++	++	+	+	++	+	++
Detection Bias	6. Can we be confident in the exposure characterization?	++	++	++	++	++	++	++	++	-	++	++	++	++	++	++	-
	7. Can we be confident in the outcome assessment?	-	-	-	-	++	-	++	-	++	++	-	-	++	++	++	++
Selective Reporting Bias	8. Were all measured outcomes reported?	++	+	++	++	+	++	+	++	++	++	++	++	++	-	++	++
Other Sources of Bias	9. Were there no other potential threats to internal validity (e.g., statistical methods were appropriate and researchers adhered to the study protocol)?	+	+	+	+	+	+	+	+	+	+	+	+	+	+	+	+
Overall Tier		2	1	1	1	1	2	1	1	1	1	1	1	1	2	1	2

++ Definitely low risk of bias

\+ Probably low risk of bias

\- Probably high risk of bias

\- - Definitely high risk of bias

Tier 1 = low risk, Tier 2 = moderate, Tier 3 = high risk of bias

Table S4. The risk of bias assessment and tier classifications of the included clinical trials on the effects of spirulina supplementation on ulcerative colitis

CATEGORY	QUESTIONS	Moradi S, 2024[11]	Moradi S, 2023[30]	Moradi S, 2023[31]
Selection Bias	Random sequence generation	+	+	+
	Allocation concealment	?	?	?
Performance Bias	Blinding of participants and personnel	+	+	+
Attrition Bias	Incomplete outcome data addressed	+	+	+
Detection Bias	Blinding of outcome assessment	+	+	+
Reporting Bias	Selective reporting	-	+	+
Other Sources of Bias	Anything else	+	+	+
Overall Tier				

\+ Low risk of bias; ? Unclear risk of bias; - High risk of bias

-

Table S5. Stratified meta-analysis of the effects of spirulina on clinical and histopathological features in ulcerative colitis

**Variable**		**Subgroups**	**Number of effect sizes**	**Pooled effect size (95% CI)**	**P-for-difference**	**P-for-heterogeneity**	**I^2 (%)**
**Weight change**	All		19	8.61 (2.98 to 14.25)	<0.001	<0.001	99.78
	Type of animals	*Rat*	9	14.35 (3.52 to 25.18)	0.04	< 0.001	97.84
		Mice	10	2.90 (1.90 to 3.91)		< 0.001	80.65
	Spirulina form	*Whole*	13	3.79 (0.68 to 6.90)	0.05	< 0.001	98.58
		Extract	6	17.91 (4.29 to 31.53)		< 0.001	99.70
	UC model	*AA*	5	11.07 (-2.95 to 25.10)	0.11	< 0.001	96.24
		DSS	10	8.25 (0.22 to 16.28)		< 0.001	99.82
		Others	4	1.54 (1.20 to 1.88)		0.29	0.00
	ROB	*Low risk*	15	9.97 (3.51 to 16.43)	0.33	< 0.001	99.72
		Moderate risk	4	3.33 (-8.33 to 14.99)		< 0.001	91.59
**Disease activity index**	All		10	-2.39 (-2.95 to -1.83)	<0.001	0.21	5.89
	Type of animals	*Rat*	6	-2.37 (-3.04 to -1.70)	0.61	0.49	0.00
		Mice	4	-2.83 (-4.49 to -1.18)		0.05	63.96
	Spirulina form	*Whole*	6	-2.55 (-3.26 to -1.84)	0.80	0.45	0.00
		Extract	4	-2.36 (-3.63 to -1.09)		0.08	51.23
	ROB	*Low risk*	8	-2.19 (-2.77 to -1.60)	0.09	0.24	0.00
		Moderate risk	2	-3.59 (-5.08 to -2.09)		0.93	0.00
**Colon length**	All		11	1.25 (0.59 to 1.91)	<0.001	<0.001	95.80
	Type of animals	*Rat*	2	2.74 (0.80 to 4.69)	0.09	0.01	84.08
		Mice	9	0.97 (0.40 to 1.54)		< 0.001	94.32
	Spirulina form	*Whole*	7	0.78 (0.59 to 0.98)	0.14	0.67	0.00
		Extract	4	1.99 (0.40 to 3.59)		< 0.001	98.69
**Histopathological score**	All		9	-1.89 (-4.51 to 0.73)	0.16	<0.001	94.31
	Type of animals	*Rat*	2	-2.00 (-3.07 to -0.94)	0.97	0.44	0.00
		Mice	7	-1.93 (-5.62 to 1.76)		<0.001	95.61
	Spirulina form	*Whole*	7	-1.22 (-4.04 to 1.60)	0.38	<0.001	93.79
		Extract	2	-5.56 (-14.91 to 3.79)		0.01	86.15
	ROB	*Low risk*	7	-1.93 (-5.62 to 1.76)	0.97	<0.001	95.61
		Moderate risk	2	-2.00 (-3.07 to -0.94)		0.44	0.00

Abbreviations: AA: acetic acid; CI: confidence Interval; DSS: dextran sulfate sodium; ROB: risk of bias; UC: ulcerative colitis

Table S6. Stratified meta-analysis of the effects of spirulina on oxidative stress in ulcerative colitis

Variable		Subgroups	Number of effect sizes	Pooled effect size (95% CI)	P-for-difference	P-for-heterogeneity	I^2 (%)
**Colonic MDA**	All		8	-4.47 (-6.52 to -2.42)	<0.001	<0.001	82.62
	Spirulina form	*Whole*	6	-3.54 (-5.42 to -1.65)	**0.04**	<0.001	79.50
		Extract	2	-7.81 (-11.48 to -4.14)		0.28	13.55
	ROB	*Low risk*	4	-6.75 (-10.79 to -2.72)	0.11	<0.001	76.80
		Moderate risk	4	-3.05 (-5.03 to -1.06)		<0.001	74.88
**Colonic SOD**	All		4	4.02 (2.77 to 5.28)	<0.001	0.56	0.00
	Spirulina form	*Whole*	2	4.08 (2.36 to 5.81)	0.81	0.98	0.00
		Extract	2	4.54 (1.28 to 7.81)		0.15	50.79
	ROB	*Low risk*	2	4.54 (1.28 to 7.81)	0.81	0.15	0.00
		Moderate risk	2	4.08 (2.36 to 5.81)		0.98	50.79
**Colonic catalase**	All		6	2.40 (0.15 to 4.66)	0.04	<0.001	90.50
	Spirulina form	*Whole*	4	1.43 (-0.72 to 3.58)	0.27	<0.001	89.66
		Extract	2	8.06 (-3.45 to 19.57)		0.01	83.38
	ROB	*Low risk*	3	4.94 (-2.10 to 11.98)	0.42	<0.001	96.65
		Moderate risk	3	1.76 (-1.44 to 4.97)		<0.001	91.82

Abbreviations: CI: confidence Interval; MDA: malondialdehyde; ROB: risk of bias; SOD: superoxide dismutase; UC: ulcerative colitis

Table S7. Stratified meta-analysis of the effects of spirulina on inflammatory markers in ulcerative colitis

Variable		Subgroups	Number of effect sizes	Pooled effect size (95% CI)	P-for-difference	P-for-heterogeneity	I^2 (%)
**Colonic TNF-α**	All		8	-4.78 (-6.47 to -3.10)	<0.001	0.01	60.41
	Type of animals	*Rat*	3	-5.13 (-8.21 to -2.05)	0.86	0.05	67.27
		Mice	5	-4.78 (-7.12 to -2.44)		0.03	59.85
	Spirulina form	*Whole*	4	-5.26 (-7.94 to -2.58)	0.74	0.10	51.46
		Extract	4	-4.62 (-7.141 to -2.11)		0.02	69.00
	UC model	*AA*	2	-3.89 (-6.28 to -1.50)	0.28	0.17	46.69
		DSS	4	-4.87 (-7.79 to -1.96)		0.02	71.28
		Others	2	-7.21 (-10.54 to -3.88)		0.37	0.00
	ROB	*Low risk*	6	-4.75 (-6.64 to -2.85)	0.83	0.03	53.61
		Moderate risk	2	-5.41 (-11.06 to 0.23)		0.02	80.28
**Colonic IL-6**	All		7	-4.56 (-7.35 to -1.77)	<0.001	<0.001	88.18
	Type of animals	*Rat*	2	-5.49 (-13.48 to 2.05)	0.80	<0.001	88.14
		Mice	5	-4.38 (-7.64 to -1.12)		<0.001	81.63
	Spirulina form	*Whole*	3	-3.78(-6.78 to -0.77)	0.61	0.08	58.45
		Extract	4	-5.22 (-9.96 to -0.48)		<0.001	89.45
**Colonic IL-1β**	All		5	-5.81 (-8.88 to -2.75)	<0.001	<0.001	76.86
	Type of animals	*Rat*	3	-6.13 (-11.53 to -0.73)	0.92	<0.001	84.81
		Mice	2	-5.83 (-8.28 to -3.37)		0.63	0.00
	Spirulina form	*Whole*	2	-4.49 (-10.91 to 1.92)	0.54	<0.001	87.41
		Extract	3	-6.62 (-8.961 to -4.28)		0.32	5.95
	ROB	*Low risk*	3	-6.62 (-8.961 to -4.28)	0.54	0.32	5.95
		Moderate risk	2	-4.49 (-10.91 to 1.92)		<0.001	87.41
**Colonic MPO**	All		8	-2.79 (-4.29 to -1.29)	<0.001	<0.001	83.48
	Type of animals	*Rat*	5	-3.19 (-4.60 to -1.77)	0.93	0.04	61.99
		Mice	3	-3.46 (-9.54 to 2.61)		0.01	97.93
	Spirulina form	*Whole*	2	-2.71 (-3.93 to -1.49)	0.71	0.85	0.00
		Extract	6	-3.22 (-5.67 to -0.77)		<0.001	91.77
	UC model	*AA*	3	-3.00 (-4.09 to -1.92)	0.88	0.58	0.00
		DSS	5	-3.26 (-6.44 to -0.07)		<0.001	94.74
**Serum TNF-α**	All		6	-2.85 (-3.93 to -1.78)	<0.001	0.03	43.16
	Type of animals	*Rat*	4	-2.65 (-3.84 to -1.46)	0.39	0.10	50.39
		Mice	2	-6.18 (-14.14 to 1.78)		0.02	80.11
	Spirulina form	*Whole*	3	-2.03 (-2.91 to -1.14)	0.08	0.74	0.00
		Extract	3	-5.08 (-8.35 to -1.82)		0.05	71.73
	UC model	*AA*	2	-1.86 (-2.84 to -0.88)	0.04	0.90	0.00
		DSS	4	-3.86 (-5.46 to -2.26)		0.07	37.95
**Serum IL-6**	All		6	-8.66 (-16.03 to -1.29)	0.02	<0.001	97.65
	Type of animals	*Rat*	4	-7.70 (-15.61 to 0.20)	0.66	<0.001	97.02
		Mice	2	-13.77 (-39.30 to 11.76)		<0.001	88.10
	Spirulina form	*Whole*	3	-3.44 (-5.41 to -1.47)	0.11	0.11	56.29
		Extract	3	-16.39 (-32.33 to -0.44)		<0.001	89.86
	UC model	*AA*	2	-4.43 (-7.50 to -1.36)	0.24	0.11	59.88
		DSS	4	-12.20 (-24.88 to 0.47)		<0.001	98.52
**Serum IL-1β**	All		4	-5.01 (-8.78 to -1.23)	0.01	<0.001	85.39
	Type of animals	*Rat*	2	-5.02 (-7.72 to -2.32)	0.84	0.20	39.26
		Mice	2	-6.14 (-16.89 to 4.60)		0.01	86.82

	UC model	*AA*	2	-5.02 (-7.72 to -2.32)	0.84	0.20	39.26
		DSS	2	-6.14 (-16.89 to 4.60)		0.01	86.82

Abbreviations: AA: acetic acid; CI: confidence Interval; DSS: dextran sulfate sodium; IL: interleukin; MPO: myeloperoxidase; ROB: risk of bias; TNF-α: tumor necrosis factor-alpha; UC: ulcerative colitis

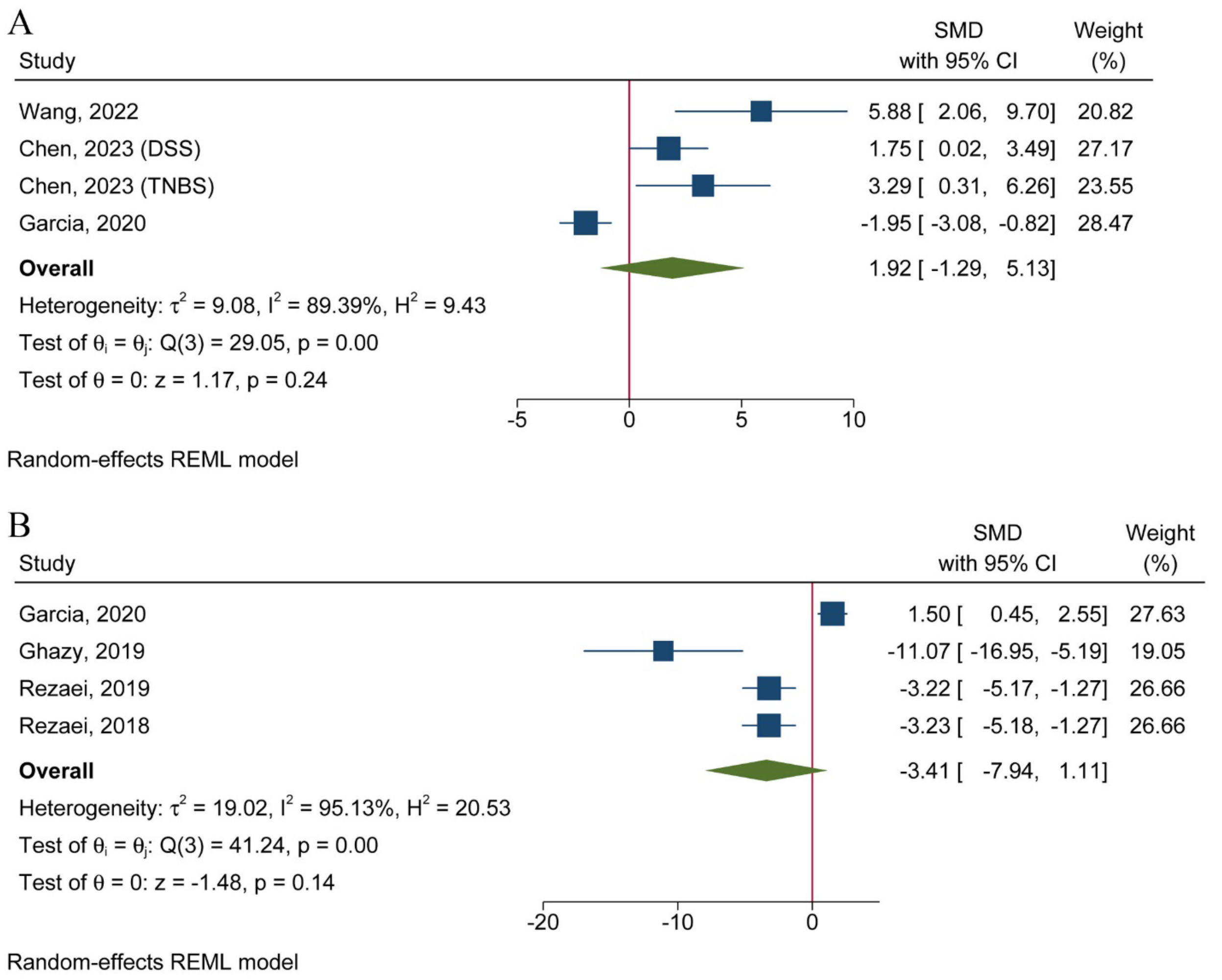

Figure S1. Forest plot depicting the effects of spirulina on colonic interleukin-10 (A) and nitric oxide (B) in ulcerative colitis

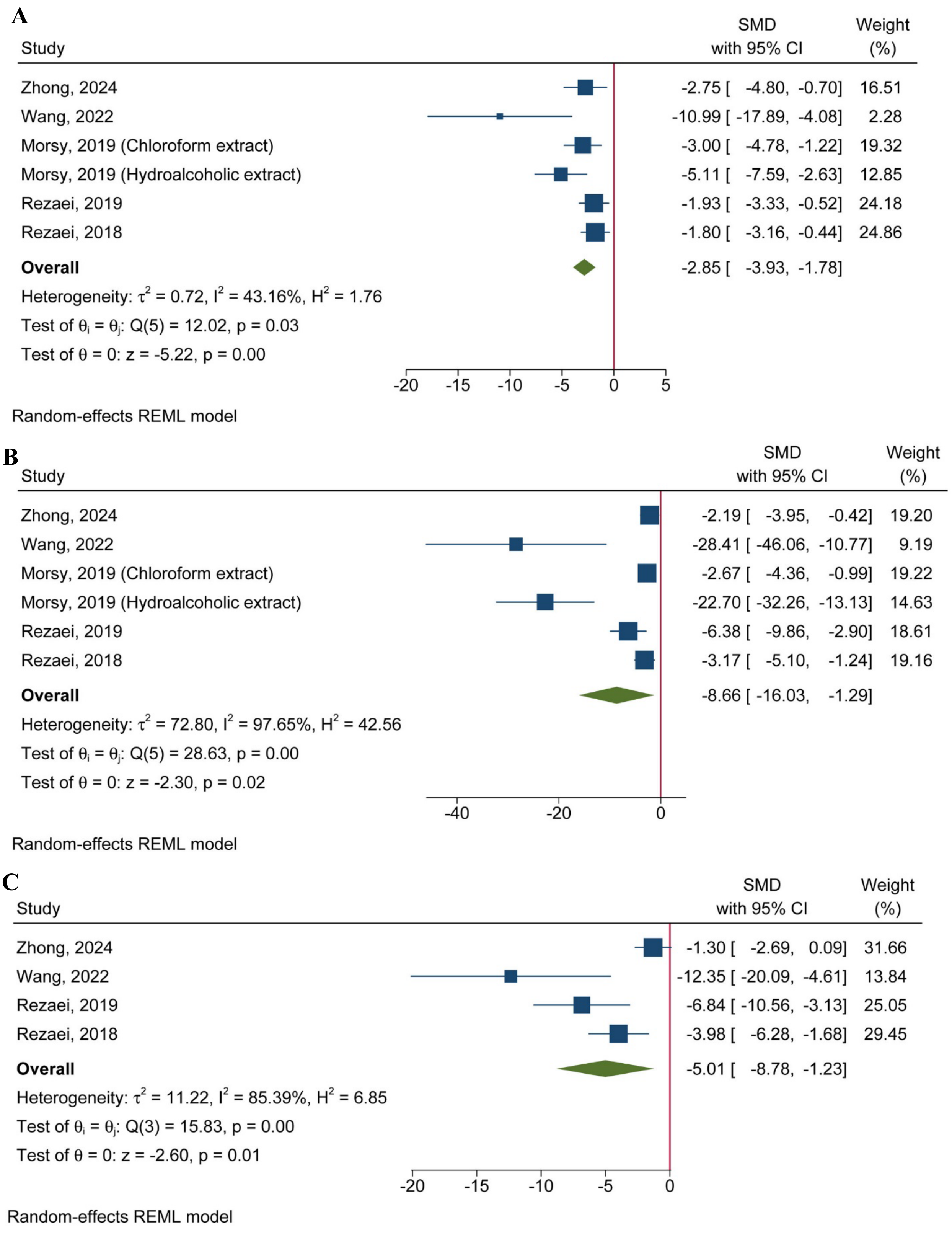

Figure S2. Forest plot depicting the effects of spirulina on the serum levels of inflammatory markers: tumor necrosis factor-α (A), interleukin-6 (B), and interleukin-1β (C) in ulcerative colitis

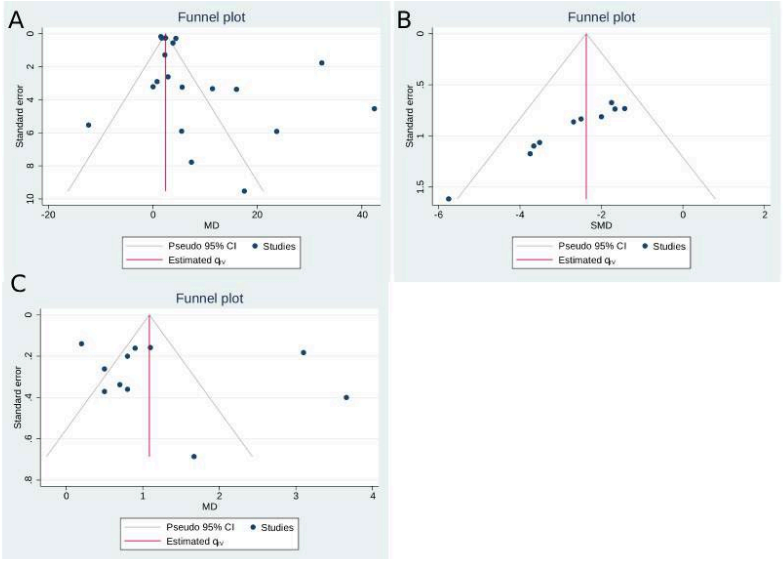

Figure S3. Funnel plots for the effects of spirulina on body weight (A), disease activity index (B), and colon length (C) in ulcerative colitis

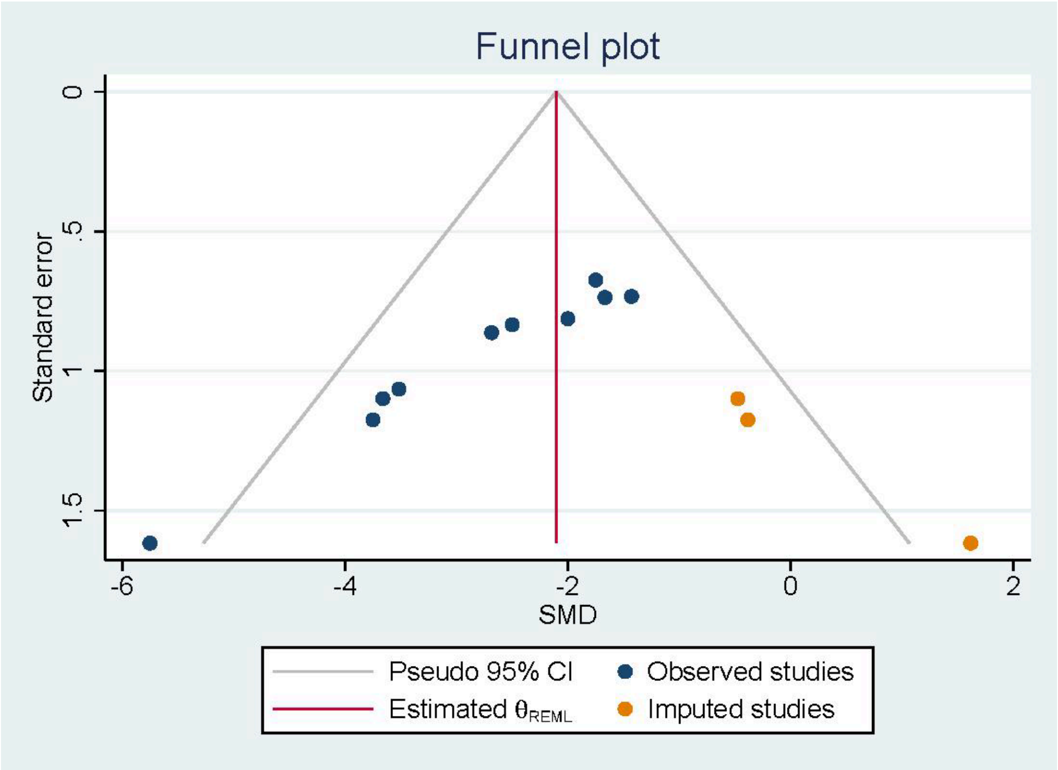

Figure S4. Trim and fill analysis for the effects of spirulina on disease activity index in ulcerative colitis
